# Genetic Architecture of Ischaemic Strokes after COVID-19 Shows Similarities with Large Vessel Strokes

**DOI:** 10.3390/ijms241713452

**Published:** 2023-08-30

**Authors:** Laia Llucià-Carol, Elena Muiño, Natalia Cullell, Jara Cárcel-Márquez, Miquel Lledós, Cristina Gallego-Fabrega, Jesús Martin-Campos, Joan Martí-Fàbregas, Ana Aguilera-Simón, Anna M. Planas, Marta L. DeDiego, Alicia de Felipe Mimbrera, Jaime Masjuan, Sebastián García-Madrona, Tomás Segura, Esther González-Villar, Gemma Serrano-Heras, Ana Domínguez Mayoral, Paloma Menéndez-Valladares, Joan Montaner, Isabelle Migeotte, Souad Rahmouni, Gilles Darcis, David Bernardo, Silvia Rojo, Eva C. Schulte, Ulrike Protzer, Lisa Fricke, Christof Winter, Mari E. K. Niemi, Mattia Cordioli, Pilar Delgado, Israel Fernández-Cadenas

**Affiliations:** 1 Stroke Pharmacogenomics and Genetics, Institut d’Investigació Biomèdica Sant Pau (IIB SANT PAU), Sant Quintí 77-79, 08041 Barcelona, Spain; lllucia@santpau.cat (L.L.-C.); mlledos@santpau.cat (M.L.);; 2 Departament de Genètica i de Microbiologia, Universitat Autònoma de Barcelona, 08193 Bellaterra, Spain; 3 Department of Neurology, Hospital Universitari MútuaTerrassa, Fundació Docència i Recerca MútuaTerrassa, 08221 Terrassa, Spain; 4 Department of Neurology, Institut d’Investigació Biomèdica Sant Pau (IIB SANT PAU), Hospital de la Santa Creu i Sant Pau, 08041 Barcelona, Spain; 5 Institute for Biomedical Research of Barcelona (IIBB), Spanish National Research Council (CSIC), 08036 Barcelona, Spain; 6 Institut d’Investigacions Biomèdiques August Pi i Sunyer (IDIBAPS), 08036 Barcelona, Spain; 7 Department of Molecular and Cell Biology, Centro Nacional de Biotecnología (CNB-CSIC), Campus Universidad Autónoma de Madrid, 28049 Madrid, Spain; marta.lopez@cnb.csic.es; 8 Instituto Ramón y Cajal de Investigación Sanitaria, Hospital Universitario Ramón Y Cajal, 28034 Madrid, Spain; 9 Department of Neurology, University Hospital of Albacete, 02006 Albacete, Spain; 10 Institute of Biomedicine of Seville (IBiS), Hospital Universitario Virgen del Rocío, CSIC, Universidad de Sevilla, 410113 Seville, Spain; 11 Department of Neurology, Hospital Universitario Virgen Macarena, 41009 Seville, Spain; 12 Fonds de la Recherche Scientifique (FNRS), 1000 Brussels, Belgium; 13 Centre de Génétique Humaine, Hopital Erasme, Université Libre de Bruxelles, 1050 Brussels, Belgium; 14 Department of Biomedical and Preclinical Sciences, Faculty of Medicine, GIGA-Insitute, University of Liege, 4000 Liège, Belgium; 15 CHU of Liege, 4000 Liège, Belgium; 16 Mucosal Immunology Lab, Unidad de Excelencia del Instituto de Biomedicina y Genética Molecular (IBGM), Universidad de Valladolid-CSIC, 47005 Valladolid, Spain; 17 Department of Microbiology, Hospital Clínico Universitario de Valladolid, Gerencia Regional de Salud de Castilla y León (SACYL), 47003 Valladolid, Spain; 18 Institute of Virology, Technical University Munich/Helmholtz Zentrum München, 81377 Munich, Germany; 19 Institute of Psychiatric Phenomics and Genomics, University Hospital, LMU Munich University, 80336 Munich, Germany; 20 Department of Psychiatry, University Hospital, LMU Munich University, 80336 Munich, Germany; 21 Institute of Human Genetics, University Hospital Bonn, Medical Faculty, University of Bonn, 53127 Bonn, Germany; 22 Department of Psychiatry and Psychotherapy, University Hospital Bonn, Medical Faculty, University of Bonn, 53127 Bonn, Germany; 23 Department of Internal Medicine II, Klinikum Rechts der Isar, School of Medicine, Technical University of Munich (TUM), 81675 Munich, Germany; lisa.fricke@mri.tum.de; 24 Institute of Clinical Chemistry and Pathobiochemistry, School of Medicine, Technische Universität München (TUM), 81675 Munich, Germany; 25 TranslaTUM, Center for Translational Cancer Research, Technische Universität München, 81675 Munich, Germany; 26 Institute for Molecular Medicine Finland (FIMM), University of Helsinki, 00014 Helsinki, Finland; mari.niemi@novartis.com (M.E.K.N.);; 27 Department of Neurology, Hospital Universitari de la Vall d’Hebrón, Universitat Autònoma de Barcelona, 08035 Barcelona, Spain

**Keywords:** COVID-19, ischaemic stroke, GWAS, local genetic correlation, PRS

## Abstract

We aimed to analyse whether patients with ischaemic stroke (IS) occurring within eight days after the onset of COVID-19 (IS-COV) are associated with a specific aetiology of IS. We used SUPERGNOVA to identify genome regions that correlate between the IS-COV cohort (73 IS-COV cases vs. 701 population controls) and different aetiological subtypes. Polygenic risk scores (PRSs) for each subtype were generated and tested in the IS-COV cohort using PRSice-2 and PLINK to find genetic associations. Both analyses used the IS-COV cohort and GWAS from MEGASTROKE (67,162 stroke patients vs. 454,450 population controls), GIGASTROKE (110,182 vs. 1,503,898), and the NINDS Stroke Genetics Network (16,851 vs. 32,473). Three genomic regions were associated (*p*-value < 0.05) with large artery atherosclerosis (LAA) and cardioembolic stroke (CES). We found four loci targeting the genes *PITX2* (rs10033464, IS-COV beta = 0.04, *p*-value = 2.3 × 10^−2^, se = 0.02), previously associated with CES, *HS6ST1* (rs4662630, IS-COV beta = −0.04, *p*-value = 1.3 × 10^−3^, se = 0.01), *TMEM132E* (rs12941838 IS-COV beta = 0.05, *p*-value = 3.6 × 10^−4^, se = 0.01), and *RFFL* (rs797989 IS-COV beta = 0.03, *p*-value = 1.0 × 10^−2^, se = 0.01). A statistically significant PRS was observed for LAA. Our results suggest that IS-COV cases are genetically similar to LAA and CES subtypes. Larger cohorts are needed to assess if the genetic factors in IS-COV cases are shared with the general population or specific to viral infection.

## 1. Introduction

Coronavirus disease (COVID-19) is a worldwide contagious and infectious disease caused by the severe acute respiratory syndrome coronavirus 2 (SARS-CoV-2). As the pandemic progressed, increased rates of thrombotic events were reported in patients with COVID-19 [1], especially ischaemic stroke (IS) [2,3]. During 2020, the incidence rate varied between 0.9% and 2.5% in different cohorts of European and Asian ancestry populations [1,2,3,4,5], whereas the incidence in the general population is 0.095% [6]. This incidence variation in COVID-19 cases is probably due to differences in the severity of COVID-19, the prevalence of vascular risk factors, including age, male gender, hypertension, hyperlipidaemia, ischaemic heart disease, diabetes mellitus type 1, the ability to accurately diagnose all strokes in a situation of saturation of medical services, and methodological differences in the studies [7,8]. However, COVID-19 patients have an approximately sevenfold higher risk of stroke compared to influenza patients [9]. Furthermore, strokes tend to be more severe and have a higher mortality in SARS-CoV-2 patients compared to those without this condition [8,10]. Moreover, in a study of 1,595,984 patients, it was concluded that those who had recovered from COVID-19 had a higher risk of suffering a stroke than the general population during the subsequent 9 months, with 4.40 per 1000 patients experiencing a stroke compared to 3.23 per 1000 patients in the control group [11].

With regards to the TOAST (Trial of ORG 10172 in acute stroke treatment) classification [12], it appears that patients with COVID-19 are predisposed to have large artery atherosclerosis (LAA) strokes [13,14], although some studies suggest that undetermined (UND) [15], cryptogenic [5,16], and cardioembolic (CES) aetiologies [15] may comprise the highest proportion. In a cohort of 32 stroke cases due to COVID-19, 65% were classified as cryptogenic [5]. Similarly, in another study of 129 cases, the percentage of cryptogenic strokes was 42% [16]. In another cohort of 91 cases, 33% were classified as CES and 34% as UND [15].

The pro-inflammatory response caused by the cytokine and chemokine storm during infection may lead to various complications, including hypercoagulability, endothelial damage, vasculitis, and thrombosis, thus leading to strokes [17]. In severe COVID-19 cases, patients often exhibit thrombocytopenia and elevated D-dimer, which in turn are associated with high levels of fibrin degradation products and low antithrombin activity, indicating changes in blood coagulation [18]. There is also evidence suggesting that COVID-19 triggers the release of matrix metalloproteinases, which cleave tight junction proteins, promoting damage to the endothelium and increasing the permeability of the blood–brain barrier. This leads to astrocyte dysfunction and activation of the inflammasome, which may contribute to an imbalance in the coagulation system [19]. Likewise, reduced functioning of the virus’s cellular entry receptor, the angiotensin-converting enzyme-2 (ACE-2) receptor, would increase angiotensin II formation, resulting in a prothrombotic state and vasoconstriction and increasing the risk of IS [20]. Additionally, COVID-19-related cardiac complications, such as tachyarrhythmia, myocardial infarction, cardiomyopathy, or changes in the intravascular volume due to infection, might alter cerebral perfusion pressures or increase atrial fibrillation, a major cause of cardioembolic strokes [7]. Therefore, the intense inflammatory response combined with a haemostatic disorder, characterized by hypercoagulable states and cardiac complications, may act as triggers for blood clot formation [19]. Nevertheless, it is essential to consider other intrinsic mechanisms related to viral infection rather than only a generalized response to severe diseases [13]. For example, the damage to the endothelial cells is directly exacerbated by the SARS-CoV-2 virus [19].

An incomplete aetiological evaluation of stroke patients with COVID-19 may be a significant confounding factor in treatment. As such, genetics may be informative in classifying IS-COV. Indeed, genetic liability for COVID-19 severity and susceptibility are associated with risk for IS [13,14]. As genetic factors depend on stroke aetiology [21,22,23], we aimed to determine whether IS due to COVID-19 (IS-COV) genetically resembles a particular subtype of IS. This investigation would offer valuable insights into whether these ischaemic strokes are attributed to underlying risk factors or directly caused by the viral infection. It would also provide a comprehensive understanding of the biological mechanisms underlying stroke and its pathogenesis.

## 2. Results

### 2.1. Local Genetic Covariance Estimation

We used SUPERGNOVA to estimate local and global genetic correlations between IS-COV and different subtypes of ischaemic stroke. We utilized GWAS data from the MEGASTROKE, GIGASTROKE, and SiGN datasets, including all ischaemic stroke (AIS), small vessel occlusion (SVO) stroke LAA, and CES. Additionally, we incorporated GWAS data for UND from the SiGN study. We partitioned the genome into 2186 independent regions using LDetect [24], with LD estimated from the 1000 Genomes Project phase III samples of European ancestry [25].

We identified thirty-one statistically significant regions (*p*-value < 0.05) that correlated between IS-COV and the different types of ischaemic stroke: five for AIS, eight for LAA, four for CES, seven for SVO, four for AIS and CES, two for AIS and LAA, and one for UND and SVO (Supplementary Table S3). 

**Figure 1:**
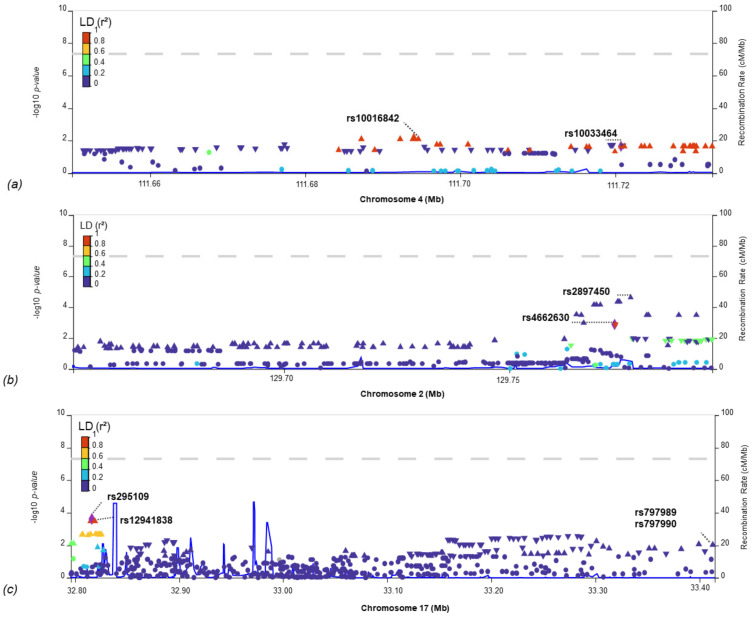
LocusZoom image of the shared region between IS-COV and the study phenotype: (**a**) CES; (**b**,**c**) LAA. The single-nucleotide variants with the most significant *p*-value in the IS-COV GWAS, as well as the most significant SNV in the IS-COV GWAS but shared in MEGASTROKE, GIGASTROKE, or SIGN.

None of these regions reached the Bonferroni threshold (*p*-value < 0.05/(2186 × 13) = 1.4 × 10^−5^), where 2186 represents the number of independent regions into which the genome is divided, and 13 is the number of comparisons made. However, we prioritized two genomic regions on chromosomes two and seventeen associated with LAA and one on chromosome four associated with CES that were consistent (*p*-value < 0.05 and correlation in the same direction) in all MEGASTROKE [26], GIGASTROKE [23], and SiGN [21] studies (Table 1). To prioritise genes in these three associated regions, we examined them using LocusZoom [27] and using V2G score [28]. For chromosome 17, LocusZoom highlighted the genes *TMEM132E, RFFL, CCT6B, ZNF830, LIG3*, and *AC004223.3* (Figure 1). We also selected SNVs with a *p*-value < 0.05 in each GWAS pair tested. Next, we focused on studying the most significant SNV in the IS-COV cohort for each locus. These SNVs are rs10033464 (beta 0.04, *p*-value 2.3 × 10^−2^, se 0.02), rs4662630 (beta −0.04, *p*-value 1.3 × 10^−3^, se 0.01), rs12941838 (beta 0.05, *p*-value 3.6 × 10^−4^, se 0.01), and rs797989 (beta 0.03, *p*-value 1.0 × 10^−2^, se 0.01; see Table 2). According to the V2G score, the score-annotated genes are *PITX2* (rs10033464), *HS6ST1* (rs4662630), *TMEM132E* (rs12941838), and *RFFL* (rs797989; see Supplementary Table S4).

### 2.2. Polygenic Risk Score

**Figure 2:**
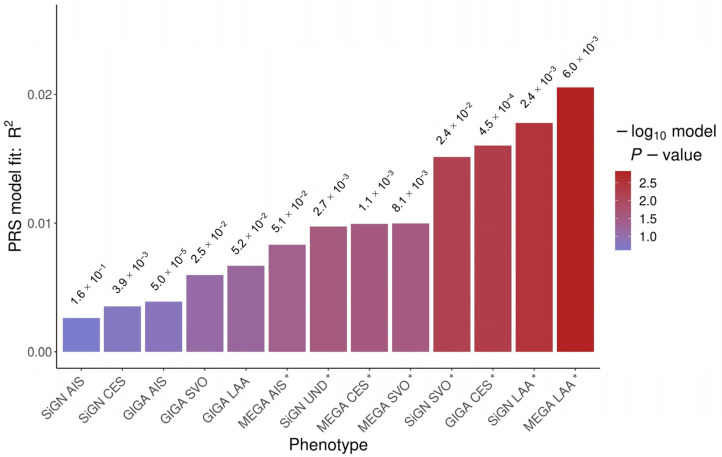
Best R^2^ bar plot for each phenotype. The *p*-value threshold used to select single-nucleotide variants (SNVs) for each PRS is shown above each bar. GIGA: GIGASTROKE; MEGA: MEGASTROKE. * PRSs that are statistically significant (*p*-value < 0.05).

For each phenotype associated with the different types of IS (AIS, CES, SVO, LAA, UND), we found at least one statistically significant PRS (*p*-value < 0.05) for the IS-COV cohort (Figure 2). 

However, only two PRSs remain statistically significant after applying the Bonferroni correction (*p*-value < 0.05/13 = 3.8 × 10^−3^). The PRS that explains the largest proportion of the variance (r^2^ = 2.1 × 10^−2^) is MEGASTROKE -LAA, with a *p*-value threshold of 6.0 × 10^−3^ and PRS *p*-value of 1.5 × 10^−3^, comprising a total of 4004 SNVs (Table 3). The second significant PRS is SiGN-LAA, with a *p*-value threshold of 2.4 × 10^−3^, an r^2^ value of 1.8 × 10^−2^, a *p*-value for the PRS of 3.2 × 10^−3^, and a total of 1305 SNVs. Both MEGASTROKE-LAA (*p*-value threshold = 6.0 × 10^−3^; r^2^= 3.4 × 10^−3^; *p*-value of the PRS = 2.2 × 10^−2^) and SiGN-LAA (*p*-value threshold = 2.4 × 10^−3^; r^2^ = 4.7 × 10^−3^; *p*-value of the PRS = 7.1 × 10^−3^) PRSs were also statically significant (*p*-value < 0.05) with PLINK 2.0 (Table 3).

## 3. Discussion

The reason for a higher frequency of strokes after COVID-19 and the aetiology of these strokes is controversial. Determining the ischaemic stroke subtype is important for secondary prevention in order to use the most appropriate treatment for each subtype. For example, for LAA, antiplatelet medications or statins may be prescribed to reduce the risk of recurrence, while for CES, anticoagulants may be considered. Furthermore, understanding the subtype of ischaemic stroke can assist in identifying risk factors that require attention to prevent future occurrences, such as atrial fibrillation in cardioembolic strokes. Moreover, the subtype of ischaemic stroke offers insights into the long-term prognosis and potential associated complications. Certain subtypes, notably cardioembolic strokes, may present a higher risk of recurrence, which can significantly impact treatment strategies and follow-up care [29]. 

In our multicentre study, we used genetics to find which subtype of ischaemic stroke is most similar to those which occurred during COVID-19 disease. Moreover, knowing the type of aetiology will contribute to a better understanding of the mechanisms underlying IS-COV to prevent stroke occurrence after COVID-19. There is increasing evidence that acute bacterial and viral infections, or chronic exposure to common infections such as influenza viruses, are risk factors for ischaemic stroke [9]. Previous genetic studies have already associated the severity of COVID-19 with the risk of ischaemic stroke [14,30] and the susceptibility to SARS-CoV-2 infection with LAA [13]. However, those studies were conducted using genetic data from patients who had suffered from either COVID-19 or ischaemic stroke but not in a specific cohort of patients who have suffered a stroke during SARS-CoV-2 infection. 

After local genetic correlations, we found three consistent regions associated with LAA and CES (Table 1). In these three regions, we found four loci targeting the genes *PITX2, HS6ST1, TMEM132E*, and *RFFL* (Table 2)*. PITX2* regulates the formation of blood vessels and the development of heart tissues [31]. Interestingly, *PITX2* and *ZFHX3* are the principal genes associated with CES [32] and atrial fibrillation [33], the most important risk factor for CES [34]. Cardiac arrhythmias and atrial fibrillation were associated with ICU admission in COVID-19 patients [35,36]. As our IS-COV patients seem to present a shared genetic susceptibility to atrial fibrillation, our hypothesis is that these patients present genetic risk factors for atrial fibrillation and CES and that this might be one of the reasons they suffer a stroke during COVID-19. 

The association between *HS6ST1, TMEM132E, RFFL*, and atherothrombotic stroke is less clear. *HS6ST1* may contribute to coagulation disorders and increase the risk of thrombotic events. The enzyme HS6ST1 catalyses the addition of a sulfate group to a specific residue of heparan sulfate (HS) molecules. HS plays an important role in regulating numerous functions, including blood coagulation and cell differentiation. Moreover, HS has anticoagulant activity by interacting with antithrombin (AT), which becomes activated and inhibits blood coagulation factors Xa and thrombin (IIa) [37]. In an ex vivo model of developing mouse neural tissue, with HS enzymatically removed by *HS6ST1* deficiency, significant suppression of Fgf8 levels was observed in murine models. Fgf8 is synthesised in the developing brain, thus suggesting its functional importance in neural development [38]. *TMEM132E* is linked to the nervous system and cellular adhesion functions. Mutations such as heterozygous variants c.382G > T: p.(Ala128Ser) and c.2204C > T: p.(Pro735Leu) [39], or the homozygous nonsense mutation Arg420Gln [40] in *TMEM132E*, have been found in patients with autosomal recessive nonsyndromic hearing loss [41]. The expression of TMEM132E was detected in the spiral ganglia of the inner ear, as well as in cranial and spinal ganglia, indicating its potential involvement in other nervous system functions beyond hearing [41]. Genetic studies have linked *TMEM132E* (rs10491113) to insomnia (rs145258459) associated with cardiometabolic diseases [42], bipolar disorder (rs10491113) [43], and panic disorder (rs887231, rs887230, and rs4795942) [44]. An in-depth structural and sequence analysis of *TMEM132* strongly predicted a cell adhesion function for the *TMEM132* family [45], and some studies have linked the role of adhesion molecules to ischaemic stroke. Finally, *RFFL* encodes for a protein that enables enzyme binding activity. *RFFL* is an important regulator of voltage-dependent hERG (human ether-a-go-go-related gene) potassium channel activity and thus cardiac repolarization [46].

Interestingly, *HS6ST1* and *RFFL* have been associated with lung diseases, namely idiopathic pulmonary fibrosis [47] and cystic fibrosis [48], respectively. *HS6T1* has also been linked to COVID-19 [49]. A cell study suggested that HS is a necessary host binding factor that promotes angiotensin-converting enzyme 2 (ACE2) binding and thus SARS-CoV-2 infection in various human cell types. The SARS-CoV-2 spike protein interacts with both cellular HS and ACE2 via its receptor-binding domain (RBD). Electron micrographs of spike protein suggest that HS enhances the open conformation of the RBD that binds ACE2. In a viral plaque assay, the inactivation of HS6ST1/2 reduced infection threefold in Hep3B cells. Accordingly, focusing drug development to treat COVID-19 on degrading, mimicking, or inhibiting HS synthesis was proposed [50].

A genetic study postulated that the genetic liability of LAA cases reported in COVID-19 patients is more likely to be intrinsic to SARS-CoV-2 infection, rather than a response associated with disease severity [13]. In this study, a PRS generated from a COVID-19 Host Genetics Initiative GWAS (36,590 COVID-19-positive cases and 1,668,938 population controls) was significantly associated with LAA from the SiGN study and MEGASTROKE GWAS using a Mendelian Randomization. Sets of co-expressed genes involved in COVID-19 susceptibility (*ISLR2* and *ACE2*) were found to be significantly enriched in LAA GWAS. These findings suggest a shared genetic background between COVID-19 susceptibility and LAA and support the hypothesis that the increased risk for LAA in COVID-19 is more closely related to the risk of SARS-CoV-2 infection than to the risk of suffering critical illness after infection [13]. These results are consistent with our findings for the *HS6ST1* gene, which has also been linked to COVID-19 susceptibility rather than COVID-19 severity. However, given that some patients with LAA already had a pre-existing atherosclerotic plaque before the SARS-CoV-2 infection, COVID-19 should be considered to often be a trigger for stroke rather than an aetiology.

Finally, we also found two PRSs associated with LAA (Table 3, Figure 2). The PRS MEGASTAROKE-LAA explains the highest proportion of phenotypic variation in our IS-COV cases. These results are consistent with the local correlations we performed. Although clinically UND is the most represented stroke subtype in IS-COV (29 cases), it is not the phenotype most genetically correlated with IS-COV. This may be due to UND GWAS having fewer cases and, therefore, less statistical power. Another possibility is that many UNDs had not completed the studies to determine the aetiology behind the stroke due to COVID-19 severity or that most of them are LAA or CES cases that could not be identified despite a complete clinical study.

One of the most important limitations of our study is the small sample size in the IS-COV cohort, even though it is the first used in this topic. This is the most probable reason for not finding any significant association with the GWAS analysis. In addition, the small sample size did not allow us to perform a stratified analysis by COVID-19 severity to determine if there are differences between COVID-19 severity and susceptibility. However, we do not have access to the TOAST classification and severity data for all IS-COV cases, as well as other clinically relevant variables necessary for characterizing the patients included in this study. Another limitation is the absence of replication for all GWAS, genetic correlation, and PRS analysis in an independent cohort of IS-COV. However, the results are consistent with the MEGASTROKE [26], GIGASTROKE [23], and SiGN [22] cohorts. Furthermore, this study does not provide sufficient evidence to establish a cause-and-effect relationship between COVID-19 and the specific aetiology of ischaemic stroke. Nevertheless, studies with a larger sample size will be necessary to establish more robust conclusions.

## 4. Materials and Methods

In this multicentre and retrospective study based on a European ancestry population, we carried out a genome-wide association study (GWAS) on COVID-19 patients who suffered an IS during the first eight days from the onset of COVID-19 symptoms vs. population controls (IS-COV cohort), as well as previously published GWASs for different phenotypes associated with IS. The data used in this study are available in the respective articles (see below) or from the corresponding author upon reasonable request. Detailed descriptions of the methods and cohorts can be found in the Supplementary Materials.

### 4.1. Cohorts’ Description

The summary statistics for AIS and four aetiology subtypes following TOAST classification (LAA, CES, SVO, UND) were obtained using the Cerebrovascular Disease Knowledge Portal (http://cerebrovascularportal.org; accessed on 22 September 2022). They were obtained from three different studies: (1) MEGASTROKE GWAS (a meta-analysis with 67,162 stroke cases and 454,450 controls) [26]; (2) GIGASTROKE (a cohort comprising 110,182 stroke patients and 1,503,898 controls) [23]; and (3) NINDS Stroke Genetics Network (SiGN with 16,851 cases and 32,473 controls) [21]. These three studies were used for all the IS (AIS), LAA, CES, and SVO data, whereas UND data were only available from the SiGN study. The number of individuals included in each GWAS used can be found in Supplementary Table S1.

The IS-COV cohort comprised 73 COVID-19 patients who suffered an IS during the first eight days since the onset of COVID-19 symptoms and 701 population controls. IS-COV controls were participants > 18 years who had not suffered from stroke or COVID-19. IS-COV cases were PCR-positive for SARS-CoV-2, aged > 18 years, and had suffered an IS during the first eight days of the infection. Detailed clinical/epidemiological data for the IS-COV cohort including age, sex, TOAST classification, and COVID-19 severity are presented in Table 4.

Most of the population controls were collected between 2003 and 2020 as a part of The CONtrol ICtus (CONIC) [51], Investigating Silent Stroke in hYpertensives: A magnetic resonance imaging Study (ISSYS) [52], and the Genotyping Recurrence Risk of Stroke (GRECOS) [53] study. In addition, IS-COV cases and additional population controls were collected between 2020 and 2021 in the Variability in immune response genes and prediction of severe SARS-CoV-2 infection (INMUNGEN-Cov2) project [54], UK Biobank [55], and the following cohorts belonging to the COVID-19 host genetics initiative [54]: Determining the Molecular Pathways and Genetic Predisposition of the COVID-19 Cohort Study of the University Medical Center of the Technical University Munich (COMRI) [56], Host genetics and immune response in SARS-CoV-2 infection/ Genetic modifiers for COVID-19 related illness (BelCovid), and Determining the Molecular Pathways and Genetic Predisposition of the Acute Inflammatory Process Caused by SARS-CoV-2 (SPGRX; see Supplementary Table S2).

### 4.2. Genotyping

DNA samples were obtained from whole blood using standard methods. Genotyping was performed using different genotyping arrays (Table 5).

### 4.3. Genotyped Data Quality Controls

Briefly, single-nucleotide variants (SNVs) that were missing in a large proportion of the subjects, non-autosomal, non-biallelic, strand ambiguous, monomorphic, or deviated from the Hardy–Weinberg equilibrium were deleted. Individuals with high rates of genotype missingness, sex discrepancy or unknown sex, family members or duplicated samples, non-European individuals, and patients with outlier heterozygosity rates were removed. Imputation was performed in the Michigan Imputation Server Pipeline [57] using the Minimac4 and HRC r1.1 2016 panel. After imputation, SNVs with an imputation score < 0.6 or minor allele frequency (MAF) < 0.01 were removed. For detailed quality controls, see the description in the Supplementary Materials. The number of patients that passed quality controls were 73 cases and 701 controls.

### 4.4. Genome-Wide Association Analysis

We performed a logistic-regression-based association analysis on the IS-COV cohort (73 cases and 701 controls) using fastGWA from GCTA [58] (Supplementary Figure S1). Age, sex, and the five principal components were used as covariates. The principal components were obtained from the imputed dosage using the gdsfmt v1.26 library. We included only independent SNVs with a genotyping rate of 0% and MAF > 10%. All SNVs with a *p*-value < 5 × 10^−8^ were considered genome-wide statistically significant.

### 4.5. Local Genetic Covariance Estimation

SUPERGNOVA (SUPER GeNetic cOVariance Analyzer) [59] is a statistical framework designed to assess the genetic correlation between two complex traits within specific regions of the genome. It utilizes summary data from the GWAS for each trait, along with the 1000 Genomes Project [25] as a reference panel, to segment the genome into independent regions by the linkage disequilibrium [36]. In our analysis, we employed GWAS data from three distinct studies (MEGASTROKE [26], GIGASTROKE [23], and SiGN [21]) to explore local genetic correlation between five phenotypes associated with the different types of IS (AIS, CES, SVO, LAA, UND) and the IS-COV phenotype.

The regions with a *p*-value < 0.05 and whose correlation goes in the same direction, and which were significant for the three data sources analysed (MEGASTROKE, GIGASTROKE, and SiGN), were considered consistent. For each locus of consistent regions, the most significant SNV in the IS-COV GWAS shared in MEGASTROKE, GIGASTROKE, or SIGN was selected using LDlink [24]. All SNVs were annotated to a gene using the Variant-to-Gene (V2G) score, which integrates experimental data from molecular phenotype quantitative trait loci, chromatin interaction, in silico functional predictions, and the distance between the variant and each gene’s canonical transcription start site [28]. In addition, we graphically evaluated the regions using LocusZoom [27].

### 4.6. Polygenic Risk Score

We utilized summary statistics for AIS, CES, SVO, LAA, and UND from MEGASTROKE [26], GIGASTROKE [23], and SiGN [21] to generate polygenic risk scores (PRSs) using the PRSice-2 [60] software and PLINK 2.0 package (https://choishingwan.github.io/PRS-Tutorial/ accessed on 18 September 2022 and 1 July 2023). PRSice-2 combines the effects of independent genetic variants identified in the GWAS and tests them in an independent cohort, in this case, the IS-COV cohort. Our aim was to determine if these single-nucleotide variants (SNVs) could significantly (*p*-value < 0.05) account for the genetic component of IS-COV.

For each GWAS summary statistic PRSice-2 generated multiple PRSs using different *p*-value thresholds of the GWAS, all adjusted for age, sex, and six principal components. These PRSs were then evaluated within the IS-COV cohort, and the optimal score threshold was selected based on the highest explained variance by the PRS (PRS r2). Each optimal score threshold was re-evaluated using PLINK 2.0.

## 5. Conclusions

Our results suggest that IS-COV cases do not resemble just one subtype of ischaemic stroke. We found that IS events due to COVID-19 genetically resemble CES and LAA subtypes. It is therefore probable that the genetic factors involved in IS-COV cases are common to genetic factors for IS in the general population. Nevertheless, the correlations we observed between LAA and IS-COV could also be intrinsic to viral infection. However, further studies with larger cohorts are needed to replicate the results, establish causality between COVID-19 and a specific subtype of ischaemic stroke, and extrapolate the results to the population.

## Figures and Tables

**Table 1:** Regions that are consistent in all analyses (*p*-value < 0.05 and correlate in the same direction).

Phenotype	Chr	Start	End	Corr	*p*-Value	SNVs
GIGA-LAA	2	129,312,188	129,864,416	0.65	1.4 × 10^−2^	198
MEGA-LAA				0.63	3.1 × 10^−2^	200
SiGN-LAA				0.99	6.0 × 10^−4^	200
GIGA-LAA	17	32,677,947	33,614,452	0.92	9.4 × 10^−3^	442
MEGA-LAA				0.91	4.6 × 10^−3^	442
SiGN-LAA				0.88	3.6 × 10^−2^	442
GIGA-CES	4	109,980,374	112,204,254	−0.87	2.1 × 10^−3^	697
MEGA-CES				−0.87	4.0 × 10^−3^	697
SiGN-CES				−0.86	6.8 × 10^−3^	697

Chr: chromosome; start: start position of the genomic region from the input genome partition file; end: end position of the genomic region from the input genome partition file; corr: estimation of local genetic correlation; *p*-value: *p*-value of local genetic covariance; SNVs: number of single-nucleotide variants involved in the estimation of local genetic covariance in the genomic region; GIGA: GIGASTROKE; MEGA: MEGASTROKE.

**Table 2:** Most significant single-nucleotide variant (SNV) in the IS-COV GWAS for each region consistent in all analyses (*p*-value < 0.05 and correlate in the same direction).

rsID	Chr	BP	A1	A2	Trait1	Trait2	*p*-Value. Trait1	B.Trait1	SE.Trait1	*p*-value. Trait2	B.Trait2	SE.Trait2
rs10033464	4	111,720,761	T	G	GIGA_CES	IS-COV	1.4 × 10^−2^	0.12	0.03	2.3 × 10^−2^	0.04	0.02
rs4662630	2	129,773,352	C	T	SiGN_LAA	IS-COV	2.7 × 10^−2^	−0.08	0.04	1.3 × 10^−3^	−0.04	0.01
rs12941838	17	32,819,326	A	G	GIGA_LAA	IS-COV	1.8 × 10^−2^	0.07	0.03	3.6 × 10^−4^	0.05	0.01
rs797989	17	33,414,758	A	C	MEGA_LAA	IS-COV	3.8 × 10^−2^	0.05	0.03	1.0 × 10^−2^	0.03	0.01

Chr: chromosome; BP: base pair position; A1: effect allele; A2: alternative allele; *p*-value.Trait: the *p*-value of the single-nucleotide variant in the GWAS; B.Trait: the effect calculated with the effect allele in the GWAS. SE.Trait: standard error; GIGA: GIGASTROKE; MEGA: MEGASTROKE.

**Table 3:** Statistically significant PRSs.

			PRSice-2		PLINK 2.0	
PRS	Threshold	Num_SNV	R^2^	*p*-Value	R^2^	*p*-Value
MEGA_LAA *	6.0 × 10^−3^	4004	2.1 × 10^−2^	1.5 × 10^−3^	3.4 × 10^−3^	2.2 × 10^−2^
SiGN_LAA *	2.4 × 10^−3^	1305	1.8 × 10^−2^	3.2 × 10^−3^	4.7 × 10^−3^	7.1 × 10^−3^
GIGA_CES	4.5 × 10^−4^	582	1.6 × 10^−2^	5.0 × 10^−3^	1.3 × 10^−3^	1.6 × 10^−1^
SiGN_SVO *	2.4 × 10^−2^	9276	1.5 × 10^−2^	6.3 × 10^−3^	3.0 × 10^−3^	3.4 × 10^−2^
MEGA_SVO	8.1 × 10^−3^	5068	1.0 × 10^−2^	2.7 × 10^−2^	1.9 × 10^−3^	8.9 × 10^−2^
MEGA_CES	1.1 × 10^−3^	1089	9.9 × 10^−3^	2.5 × 10^−2^	9.7 × 10^−4^	2.2 × 10^−1^
SiGN_UND	2.7 × 10^−3^	1453	9.7 × 10^−3^	2.6 × 10^−2^	2.5 × 10^−3^	5.2 × 10^−2^
MEGA_AIS	5.1 × 10^−2^	19,869	8.3 × 10^−3^	4.0 × 10^−2^	1.9 × 10^−3^	9.1 × 10^−2^
GIGA_LAA	5.2 × 10^−2^	21,350	6.7 × 10^−3^	6.2 × 10^−2^	5.4 × 10^−4^	3.6 × 10^−1^
GIGA_SVO	2.5 × 10^−2^	12,262	6.0 × 10^−3^	8.2 × 10^−2^	2.1 × 10^−3^	7.4 × 10^−2^
GIGA_AIS	5.0 × 10^−5^	267	3.9 × 10^−3^	1.6 × 10^−1^	4.9 × 10^−4^	3.9 × 10^−1^
SiGN_CES	3.9 × 10^−3^	2067	3.5 × 10^−3^	1.8 × 10^−1^	3.1 × 10^−6^	9.5 × 10^−1^
SiGN_AIS	1.6 × 10^−1^	39,071	2.6 × 10^−3^	2.4 × 10^−1^	4.8 × 10^−4^	3.9 × 10^−1^

Threshold: *p*-value threshold used to select SNVs; R^2^: variance explained by the PRS; *p*-value: the *p*-value of the PRS; SNVs: number of single-nucleotide variants included; GIGA: GIGASTROKE; MEGA: MEGASTROKE. * *p*-value < 0.05 in both PRSice-2 and PLINK; in green are those significant after Bonferroni correction.

**Table 4:** Descriptive table for the patients included in this study.

	Cases (N = 73)	Controls (N = 701)	Overall (N = 774)
Age			
Mean (SD)	70.6 (13.0)	65.6 (9.34)	66.1 (9.84)
Median [Min, Max]	73.0 [36.0, 90.0]	67.0 [23.0, 90.0]	67.0 [23.0, 90.0]
Sex			
Female	24 (32.9%)	352 (50.2%)	376 (48.6%)
Male	49 (67.1%)	349 (49.8%)	398 (51.4%)
	**Cases (N = 73)**		
Severity of COVID-19			
Hospitalized ICU	34 (46.6%)		
Hospitalized not ICU	13 (17.8%)		
Not hospitalized	14 (19.2%)		
Missing	12 (16.4%)		
TOAST			
CES	13 (17.8%)		
INF	4 (5.5%)		
LAA	10 (13.7%)		
SVO	5 (6.8%)		
UND	29 (39.7%)		
Missing	12 (16.4%)		

ICU: intensive care unit; CES: cardioembolic stroke; INF: infrequent aetiology; LAA: large artery atherosclerosis; SVO: small vessel occlusion; UND: undetermined aetiology.

**Table 5:** Participants from each cohort were included in this study, and different genotyping arrays were used.

Project	Array	Controls	Cases
CONIC	Illumina^®^ Human Core Exome chip	189	
ISSYS	Illumina^®^ Human Core Exome chip	274	
GRECOS	Illumina^®^ Human Core Exome chip	189	
INMUNGEN-CoV2	Axiom Spain Biobank Array	49	45
UK Biobank	Applied Biosystems UK BiLEVE Axiom and Applied Biosystems UK Biobank Axiom Array		12
BelCovid	Illumina’s Human OmniExpress BeadChips		12
SPGRX	Infinium Global Screening Array-24		2
COMRI	Infinium Global Screening Array-24 v3.0 Kit		2

## Data Availability

The datasets used in the current study are available from the corresponding author upon reasonable request.

## Supplementary Materials

The following supporting information can be downloaded at https://www.mdpi.com/article/10.3390/ijms241713452/s1.
