# Supplementary material for: Genetic Architecture of Ischaemic Strokes after COVID-19 Shows Similarities with Large Vessel Strokes
Source: Int J Mol Sci. 2023 Aug 30;24(17):13452. doi: 10.3390/ijms241713452 (PMC10487930; doi:10.3390/ijms241713452)
Supplement: Supplementary file 1 [file ijms-24-13452-s001.zip › ijms-2515810-supplementary-latest .pdf]

Supplementary Material

Supplemental Methods

For each study, IS was subdivided into different aetiological categories using the Trial of Org 10172 in Acute Stroke Treatment (TOAST) [1]. The sample sizes of each study subtype are defined in Supplementary Table 1. Further details of the cohorts are in the original publications.

Description of the studies included

GIGASTROKE

The GIGASTROKE [2] consortium, a large-scale international collaboration launched by the International Stroke Genetics Consortium, releases the cross-ancestry GWAS meta-analyses of 29 population-based cohorts or biobanks with incident stroke ascertainment and 25 clinic-based case–control studies, comprising 110,182 patients who had a stroke (five ancestries, 33% non-European) and 1,503,898 control individuals (of whom 45.5% were in longitudinal cohorts or biobanks).

MEGASTROKE

For the European ancestry analysis of MEGASTROKE [3] consortium 16 different cohorts were analysed, comprising up to 67,162 cases of ischaemic stroke and 454,450 healthy controls. Stroke was defined according to the World Health Organization (WHO) as rapidly developing signs of focal (or global) disturbance of cerebral function, lasting more than 24 hours or leading to death with no apparent cause other than that of vascular origin. Strokes were defined as ischaemic stroke (IS) or intracerebral haemorrhage (ICH) based on clinical and imaging criteria.

NINDS Stroke Genetics Network (SiGN)

The National Institute of Neurological Disorders Stroke Genetics Network (NINDS-SiGN) [2] is an international consortium that performed a GWAS with 16,851 cases and 32,473 stroke-free controls. IS cases with consistent neuroimaging and adequate clinical data to allow phenotypic classification were included from 31 cohorts. SiGN used two subtyping systems: the Causative Classification of Stroke (CCS) and the TOAST classification systems [1].

Description of the cohorts

The number of individuals each study contributes to the analysis is specified in Supplementary Table 2. From the INMUNGEN-Cov2 cohort we selected IS-COV cases and controls [4]. From COMRI [5], BelCovid, SPGRX [4] and UK Biobank [6] we selected the IS-COV cases. From CONIC [7], GRECOS [8] and ISSYS [9] we selected the IS-COV controls.

Variability in immune response genes and prediction of severe SARS-Cov-2 infection (INMUNGEN-Cov2)

The INMUNGEN-Cov2 project was financed by the Consejo Superior de Investigaciones Científicas (CSIC) and the iBioStroke project (AC19/00106; Eranet-Neuron, European research grants). Cases were PCR+ for SARS-Cov-2 (age > 18 years) and population controls were > 18 years old. The recruitment was done between April 2020 and March 2021. Population controls were obtained from Hospital de la Santa Creu i Sant Pau (Barcelona) and Hospital Universitari Vall d'Hebron (Barcelona). IS-COV cases were obtained from Hospital de la Santa Creu i Sant Pau (Barcelona), Hospital Clínic de Barcelona (Barcelona), Hospital General Universitario de Albacete (Albacete), Hospital Universitario Ramón y Cajal (Madrid) and Hospital Universitario Virgen del Rocio Y Virgen Macarena (Sevilla) [4].

COVID-19 Cohort Study of the University Medical Center of the Technical University Munich (COMRI)

COMRI is an observational study prospectively enrolling individuals with suspected or confirmed COVID-19 and treated at the University Medical Center of the Technical University Munich. Biosamples are deposited in the COVID-19 biobank of the Faculty of Medicine of the Technical University Munich. For this study, we used results from patients with PCR-confirmed SARS-CoV-2 infection recruited between January 2020 and March 2021, and available genome-wide genotyping data. COMRI and the COVID-19 biobank of the Faculty of Medicine at Technical University Munich received ethical approval from the local research ethics board (TUM 217/20, TUM 221/20S, TUM 440/20S) [5].

Host genetics and immune response in SARS-Cov-2 infection/ Genetic modifiers for COVID-19 related illness (BelCovid)

BelCovid is a cohort composed of individuals hospitalized for COVID-19 infection compared to population controls. Samples were collected for Liege (CHU of Liege and CHC Mont-Legia) between March and December 2020, and from Brussels (Hospital ERASME) from April to June 2020. The cohort was multi-ethnic and was analysed at Institute for Molecular Medicine Finland (FIMM). With the ethical approval of Hôpital Erasme and University of Liège Academic Hospital Ethics Committees [4].

Determining the Molecular Pathways and Genetic Predisposition of the Acute Inflammatory Process Caused by SARS-CoV-2 (SPGRX)

SPGRX cohort was composed of individuals with reported COVID-19 infections compared to population controls. Both cases and controls were collected between Merch and September 2020 at Hospital Clínico Universitario de Valladolid (Spain). All of them have European ancestry. The Ethics Committees of Provincial de Granada and Investigación con Medicamentos Area de Salud Valladolid provided ethical approval [4].

UK Biobank

The UK Biobank is a population-based cohort with deep genetic and phenotypic data collected on approximately 500,000 individuals since 2014 from multiple sites across the United Kingdom (aged between 40 and 69 at recruitment) [6]. A rich variety of phenotypic and health-related information is available to each participant. IS-COV cases were those with a confirmed diagnosis of SARS-CoV-2 infection at the latest 8 days before they were recorded with one of the following ICD-10 codes: ICD-10 I634 (Cerebral infarction due to embolism of cerebral arteries), I635 (Cerebral infarction due to unspecified occlusion or stenosis of cerebral arteries), I638 (Another cerebral infarction) and I639 (Cerebral infarction, unspecified).

Genotyping RECurrence Risk Of Stroke (GRECOS)

GRECOS project is a national study that aims to find genetic factors associated with the recurrence after stroke. Consecutive Caucasian patients, aged >18 years old, have a first IS and were admitted to the emergency department of 23 Spanish Hospitals. Stroke diagnosis was performed by trained neurologists and confirmed by neuroimaging. Control participants were selected from relatives of patients (wife or husband, without any consanguinity among cases and controls) and healthy volunteers visiting the same hospital for routine testing. They were >65 years of age and classified as free of neurovascular and cardiovascular history and familial history of stroke by direct interview before recruitment [8].

Investigating Silent Stroke in hYpertensives: A magnetic resonance imaging Study (ISSYS)

ISSYS is an observational prospective study in hypertensive participants to determine the prevalence of brain infarcts defined by magnetic resonance imaging and cognitive impairment. This cohort comprises 1000 non-demented individuals, aged 50 to 70 years old, and diagnosed with essential hypertension at least one year before inclusion in the ISSYS study [9].

CONtrol ICtus (CONIC)

CONIC study is a national study that recruited controls and IS cases participants in Vall d'Hebron Hospital between 2007 and 2008 [7]. All controls were older than 65 years of age and declared free of dementia, neurovascular and/or cardiovascular disease, as evaluated by self-description during a direct interview before recruitment. Subjects with a history of first and/or second-degree neurovascular disorder were also excluded from the study. The IS cases were admitted to the emergency department of a university hospital who had a documented middle cerebral artery (MCA) occlusion on transcranial Doppler ultrasonography (TCD) and received tPA in a standard 0.9-mg/kg dose (10% bolus, 90% continuous infusion during 1 hour) within 3 hours of symptom onset following National Institute of Neurological Disorders and Stroke (NINDS) recommendations.

Description of the variables

IS-COV

COVID-19 confirmed patients that suffered an IS during the first eight days of symptom onset or COVID-19 diagnosis.

Trial of ORG 10172 in Acute Stroke Treatment (TOAST)

Ischaemic Stroke Subtype based on the TOAST classification system [1]:

- Large Artery Atherosclerosis (LAA): stenosis of more than 50% of the diameter in large calibre extracranial or intracranial arteries, or when the occlusion is less than 50% but is accompanied by vascular risk factors and there is a significant reduction in blood flow.
- Cardioembolic (CES): Ischaemic stroke results from an embolism originating in the heart.
- Small Vessel Oclussion (SVO): Lacunar syndrome with or without evidence of ischaemic lesion less than 1.5 cm in diameter in the brain stem or subcortical white matter.
- Undetermined Aetiology (UND): includes patients with two or more potential causes of stroke, a negative evaluation (unknown aetiology), or incomplete evaluation.
- Infrequent Aetiology (INF): Rare causes such as non-atherosclerotic vasculopathies, hematologic -disorders, or hypercoagulable states.
- Unknown: information not available

COVID-19 severity

Severity of COVID-19:

- Hospitalized ICU (intensive care unit): The COVID-19 disease the patient to his admitted to the ICU.
- Hospitalized not ICU: The patient with COVID-19 was hospitalized but not admitted to the ICU.
- Not hospitalized: The patient did not require hospitalization as a direct result of COVID-19.

Quality Controls

INMUNGEN-Cov2, GRECOS, ISSYS and CONIC

DNA samples were obtained from whole blood and genotypes using different arrays (Table 2). Quality control was performed on genotyped data using PLINK v1.9 and v2.0 and KING v2.2.6 software separately for each the CONIC, ISSYS, GRECOS and INMUNGEN-CoV2 cohorts. Genetic variants with a call rate lower than 0.05, non-autosomal, non-biallelic, strand ambiguous, monomorphic or deviated from Hardy-Weinberg equilibrium (p-value <10-6 for controls and <10-10 for cases) were removed. We also excluded individuals with a genotype call rate lower than 95%, sex discordance, non-European ancestry, excess or loss of heterozygosity (+-4SDs) and those who were duplicated or related (PI-HAT>0.20). Imputation was done using the Michigan Imputation Server using Minimac4 with HRC r1.1 2016 (GRCh37/hg19) as the reference panel. We imputed those cohorts that had been genotyped with the same array jointly. After imputation, SNVs with an imputation score <0.6 were removed. We selected IS-COV cases and population controls. We merged these cohorts with UK Biobank, BelCovid, SPGRX and COMRI. We then excluded non-European individuals, those with excess heterozygosity (+-4SDs), those who were duplicated or with a genotype call rate lower than 95%. We also removed variants with a call rate lower than 0.05, deviated from Hardy-Weinberg equilibrium, with a MAF <0.01 or with a minor allele count (MAC) less than one.

The UK Biobank, BelCovid, SPGRX and COMRI cohorts were imputed separately, and each passed quality control. From these cohorts, we only use IS-COV cases.

UK Biobank

DNA was extracted from stored blood samples collected from participants during their visit to a UK Biobank assessment centre. Applied Biosystems UK BiLEVE Axiom and Applied Biosystems UK Biobank Axiom arrays were used for genotype the samples. The quality control pipeline was designed specifically to accommodate the large-scale dataset and can be found in the original paper. The IMPUTE4 software was used for imputation with Haplotype Reference Consortium (HRC) and the merged UK10K and 1000 Genomes phase 3 reference panels. After imputation, we selected individuals of European ancestry by using KIng software, and excluded SNVs with imputation score <0.3 and MAF <0.01. We then removed individuals with excess heterozygosity (+-4SDs) or with high missing (call rate lower than 95%). SNVs with imputation score <0.3, call rate <0.05, deviated from Hardy-Weinberg equilibrium, or with a MAF <0.01 were also excluded.

COMRI

Cases and controls were genotyped with the Infinium Global Screening Array-24 v3.0 Kit. The quality controls were performed on genotyped data using PLINK v1.9.

- Removing subjects with a 0.02 of the rate of genotype missingness.
- Removing missing genetic variants in a 0.98 proportion of individuals.
- Removing individuals with discordances between reported and genotyped sex.
- Removing duplicate samples with identity by descent (IBD) >= 0.98.
- Ancestry was defined using autosomal markers from the 1000 Genomes phase 3 panel with MAF > 0.1 intersecting with our data were used. Markers in the Major Histocompatibility Complex (chr6:25Mb-35Mb) and chromosome 8 inversions (chr8:7Mb-13Mb) were removed. PCs were calculated using 1000 Genomes as a reference panel and all samples were projected onto the 1000 Genomes PCA space. This was all computed using the Hail software. A random forest classifier-based model was used to assign ancestries to the individuals in our data. 1000 Genomes reference panel consisting of 5 super populations: EAS, EUR, AFR, AMR, SAS was used as a training dataset for the model. RandomForestClassifier from sklearn package in Python was used for the model. We used 1000 Genomes phase3 marker reference panel.

- Removing SNPs in Hardy–Weinberg disequilibrium (HWE). The threshold was a p-value <1x10-6 for controls and <1x10-10 for cases.

The cohort was imputed with TOPMed release 2 (https://imputation.biodatacatalyst.nhlbi.nih.gov/) as the reference panel. After imputation, QC was performed. We removed SNVs with allele frequency differences between our data and gnomAD calculated using Mahalanobis Distance - MD < 30.

BelCovid

Total DNA was extracted from EDTA-collected peripheral blood. Individuals were genotyped for >700 K SNPs using Illumina's Human OmniExpress BeadChips. The iScan system and the Genome Studio software follow the guidelines of the manufacturer. Variants with call rate ≤0.95, deviating from Hardy–Weinberg equilibrium ($p \leq 10{-}4$), or which were monomorphic were removed. The European ancestry was confirmed by PCA using the HapMap population as a reference. Using the real genotypes of 629,570 quality-controlled autosomal SNPs as anchors, the Sanger Imputation Services with the UK10K + 1000 Genomes Phase 3 Haplotype panels (https://imputation.sanger.ac.uk) was used to impute genotypes at autosomal variants. Indels, SNPs with MAF ≤ 0.05, deviating from Hardy-Weinberg equilibrium ($p \leq 10{-}3$), and with low imputation quality (INFO ≤ 0.4) were removed, leaving 6,019,462 high-quality SNPs.

SPGRX

Cases and controls were genotyped with the Infinium Global Screening Array-24 v3.0 Kit. The quality controls were performed on genotyped data using PLINK v2.0.

- Removing missing genetic variants in > 0.98 proportion of individuals.
- Removing subjects with > 0.02 of the rate of genotype missingness.
- Removing non-autosomal, non-biallelic, strand ambiguous, monomorphic, or duplicated SNPs.
- Removing individuals with discordances between reported and genotyped sex.
- Removing duplicate samples (Kinship Coefficient Threshold 0.055).
- Removing non-European individuals (PCA computed with a set of Ancestry Informative Markers). A set of 2512 Ancestry Informative Markers for 1000Genomes Word Populations (1KGWORLD.SPGRX.AIMs.list, Grch37) was used for computing principal components (PCs) with Plink2.
- Removing individuals with high or low heterozygosity rates (+-4SDs).
- Removing SNPs in Hardy–Weinberg disequilibrium (HWE). The threshold was a p-value <1x10-6 for controls and <1x10-10 for cases.

The cohort was imputed with the Michigan Imputation Server using Minimac4 with 1000Genomes as the reference panel. After imputation, QC was performed. We removed SNPs with imputation score (r2) <0.6 and minor allele frequency (MAF) <0.01.

Supplemental Figures

Supplementary Figure 1. Manhattan and QQ plot of the IS-COV cohort. The Manhattan plot shows SNPs represented by dots and plotted based on their genome-wide association study p-values, with the blue line indicating genome-wide suggestive (p-value < $1\times10{-}5$). The QQ plot displays the expected −log10 - p-value under the null hypothesis on the x-axis, while lambda is the median of the resulting chi-squared test statistics divided by the expected median of the chi-squared distribution under the null hypothesis. We did not observe overall inflation of p-values, with the genomic inflation factor λ being 1.

Ischemic stroke events due to COVID-19 (IS-COV)

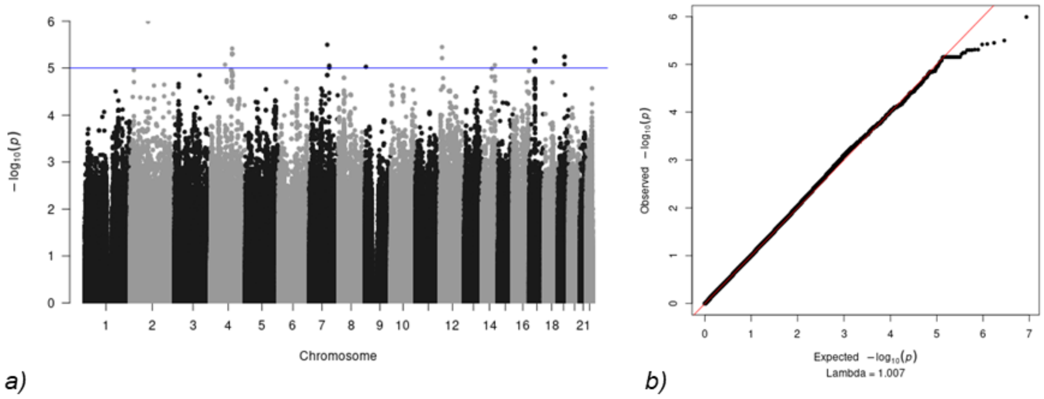

Supplemental Tables

Supplementary Table 1. Cases and controls included in each European population GWAS used in this study.

	MEGASROKE			GIGASTROKE			SiGN		
	**Casos**	**Controles**	**Total**	**Casos**	**Controles**	**Total**	**Casos**	**Controles**	**Total**
**AIS**	34217	406111	440328	62100	1234808	1296908	16851	32473	49324
**CES**	7193	355468	362661	10804	1234808	1245612	3427	32473	35900
**LAA**	4373	297290	301663	6399	1234808	1241207	2406	32473	34879
**SVO**	5386	343560	348946	6811	1234808	1241619	3186	32473	35659
**UND**							3593	32473	36066

Supplementary Table 2. The number of cases and controls from each participating hospital is detailed. *Hospitals of the INMUNGEN-Cov2 Project.

Hospital	Controls	Cases
Hospital Clínic de Barcelona (Barcelona, Spain)*		1
Hospital de la Santa Creu i Sant Pau (Barcelona, Spain)*	27	3
Hospital General Universitario de Albacete (Albacete, Spain)		12
Hospital Universitario Ramón y Cajal (Madrid, Spain)		25
Hospital Universitari Vall d'Hebron (Barcelona, Spain)	622	
Hospital Universitario Virgen del Rocio Y Virgen Macarena (Sevilla, Spain)	6	4
Hospital Universitario Río Hortega (Valladolid, Spain)	7	
Hospital del Mar (Barcelona, Spain)	6	
Hospital Universitari Germans Tries i Pujol (Badalona, Spain)	13	
Hospital Universitario de Basurto (Bilbao, Spain)	10	
Hospital Universitario Doctor Josep Trueta (Girona, Spain)	10	
Hospital Clínico Universitario de Valladolid (Valladolid, Spain)		2
University Medical Center of the Technical University Munich (Munich, Germany)		2
CHU of Liege and CHC Mont-Legia (Liège, Belgium)		12

UK Biobank (United Kingdom)		12

Supplementary Table 3. Regions with significant correlation (p-value <= 0.05) by SUPERGNOVA.

Trait1	Trait2	chr	start	end	rho	corr	h2_1	h2_2	var	p-value	SNVs
SiGN_LAA	IS_COV	4	1478711	3612028	2.5E-03	0.92	3.9E-04	1.9E-02	1.6E-06	4.6E-02	532
MEGA_CES	IS_COV	16	2294890	3960458	-9.2E-04	-0.96	3.4E-05	2.7E-02	1.6E-07	2.1E-02	401
MEGA_SVO	IS_COV	16	5855169	6264138	1.0E-03	0.88	5.0E-05	2.6E-02	2.1E-07	2.8E-02	266
MEGA_CES	IS_COV	7	18863387	19975992	-8.5E-04	-0.85	4.1E-05	2.4E-02	1.8E-07	4.4E-02	235
SiGN_AIS	IS_COV	7	18863387	19975992	-2.3E-03	-0.91	2.7E-04	2.4E-02	8.0E-07	9.5E-03	235
GIGA_LAA	IS_COV	12	22944771	23819079	4.3E-04	0.86	1.7E-05	1.4E-02	4.3E-08	4.0E-02	327
MEGA_AIS	IS_COV	14	24904240	26134815	7.9E-04	0.88	3.5E-05	2.3E-02	1.6E-07	4.5E-02	589
GIGA_LAA	IS_COV	3	27609447	29409547	-5.1E-04	-0.91	1.5E-05	2.0E-02	6.7E-08	4.8E-02	564
MEGA_LAA	IS_COV	3	27609447	29409547	-1.1E-03	-0.99	6.4E-05	2.0E-02	3.0E-07	3.8E-02	564
SiGN_AIS	IS_COV	3	27609447	29409547	-2.9E-03	-0.88	5.5E-04	2.0E-02	2.1E-06	4.5E-02	564
GIGA_SVO	IS_COV	22	27654838	29454477	-5.4E-04	-0.85	1.1E-05	3.6E-02	7.4E-08	4.6E-02	561
SiGN_UND	IS_COV	22	27654838	29454477	-3.4E-03	-0.82	4.8E-04	3.6E-02	2.4E-06	2.7E-02	561
MEGA_SVO	IS_COV	16	31384554	47981754	5.1E-04	0.99	1.6E-05	1.7E-02	5.7E-08	3.1E-02	141
SiGN_LAA	IS_COV	3	31721774	33011510	3.2E-03	0.84	7.9E-04	1.9E-02	2.1E-06	2.7E-02	324
GIGA_LAA	IS_COV	17	32677947	33614452	6.6E-04	0.92	1.9E-05	2.7E-02	6.5E-08	9.4E-03	442
MEGA_LAA	IS_COV	17	32677947	33614452	1.4E-03	0.91	9.1E-05	2.7E-02	2.5E-07	4.6E-03	442
SiGN_LAA	IS_COV	17	32677947	33614452	3.0E-03	0.88	4.3E-04	2.7E-02	2.0E-06	3.6E-02	442
GIGA_LAA	IS_COV	1	34017349	34797348	-4.2E-04	-0.91	2.0E-05	1.1E-02	3.8E-08	3.4E-02	276
MEGA_LAA	IS_COV	1	34017349	34797348	-8.0E-04	-0.98	6.4E-05	1.1E-02	1.6E-07	4.5E-02	276
GIGA_SVO	IS_COV	17	34393296	35725997	-3.1E-04	-0.96	6.7E-06	1.5E-02	1.9E-08	2.6E-02	148
MEGA_SVO	IS_COV	17	34393296	35725997	-6.2E-04	-0.88	3.2E-05	1.5E-02	7.5E-08	2.4E-02	149
MEGA_SVO	IS_COV	10	36020445	36884906	-8.5E-04	-0.89	3.3E-05	2.8E-02	1.8E-07	4.8E-02	331
GIGA_SVO	IS_COV	4	43963518	45187835	4.8E-04	0.96	9.5E-06	2.6E-02	4.1E-08	1.8E-02	396
GIGA_AIS	IS_COV	17	46828412	48027295	-4.2E-04	-0.74	2.0E-05	1.6E-02	4.6E-08	4.9E-02	325
GIGA_AIS	IS_COV	18	50152229	51059341	-4.1E-04	-0.72	1.2E-05	2.6E-02	4.2E-08	4.7E-02	369
MEGA_CES	IS_COV	18	50152229	51059341	-8.8E-04	-0.84	4.1E-05	2.6E-02	1.5E-07	2.3E-02	369
SiGN_CES	IS_COV	18	50152229	51059341	-2.6E-03	-0.83	3.5E-04	2.6E-02	1.1E-06	1.4E-02	369
GIGA_AIS	IS_COV	16	54673195	55505040	-4.8E-04	-0.93	1.2E-05	2.2E-02	5.8E-08	4.3E-02	429
GIGA_CES	IS_COV	16	54673195	55505040	-6.6E-04	-0.77	3.3E-05	2.2E-02	8.1E-08	2.1E-02	429
MEGA_LAA	IS_COV	14	75747258	76583135	7.5E-04	0.97	7.1E-05	8.5E-03	1.4E-07	4.5E-02	345
SiGN_AIS	IS_COV	6	82387920	83904869	2.4E-03	0.72	2.7E-04	4.0E-02	1.3E-06	4.1E-02	354
SiGN_CES	IS_COV	6	82387920	83904869	3.2E-03	0.95	2.8E-04	4.0E-02	1.9E-06	2.1E-02	354
GIGA_AIS	IS_COV	16	86745955	87173290	-5.8E-04	-0.99	1.6E-05	2.2E-02	4.1E-08	4.5E-03	204
GIGA_SVO	IS_COV	1	96150318	97457893	3.6E-04	0.92	9.0E-06	1.7E-02	3.2E-08	4.2E-02	332
GIGA_CES	IS_COV	4	109980374	112204254	-1.7E-03	-0.87	2.9E-04	1.3E-02	3.1E-07	2.1E-03	697
MEGA_CES	IS_COV	4	109980374	112204254	-2.8E-03	-0.87	7.9E-04	1.3E-02	9.5E-07	4.0E-03	697
SiGN_CES	IS_COV	4	109980374	112204254	-5.9E-03	-0.86	3.6E-03	1.3E-02	4.8E-06	6.8E-03	697
MEGA_CES	IS_COV	5	121485609	122603725	-9.9E-04	-0.94	5.4E-05	2.1E-02	2.5E-07	4.8E-02	298
GIGA_LAA	IS_COV	2	129312188	129864416	4.0E-04	0.65	2.4E-05	1.6E-02	2.7E-08	1.4E-02	198
MEGA_LAA	IS_COV	2	129312188	129864416	7.9E-04	0.63	1.0E-04	1.6E-02	1.3E-07	3.1E-02	200
SiGN_LAA	IS_COV	2	129312188	129864416	3.4E-03	0.99	7.6E-04	1.6E-02	9.8E-07	6.1E-04	200
GIGA_AIS	IS_COV	2	131466136	133030349	3.3E-04	0.81	1.4E-05	1.2E-02	1.7E-08	1.2E-02	157
MEGA_AIS	IS_COV	5	150918158	152360494	-6.8E-04	-1.00	6.1E-05	7.6E-03	1.2E-07	4.8E-02	474

GIGA_AIS	IS_COV	6	155229566	156756457	-9.5E-04	-0.68	3.9E-05	4.9E-02	1.6E-07	1.6E-02	575
GIGA_LAA	IS_COV	6	155229566	156756457	-7.6E-04	-0.68	2.5E-05	4.9E-02	1.3E-07	3.4E-02	575
MEGA_AIS	IS_COV	6	155229566	156756457	-1.4E-03	-0.77	6.5E-05	4.9E-02	3.4E-07	1.8E-02	575
SiGN_AIS	IS_COV	6	155229566	156756457	-4.3E-03	-0.95	4.2E-04	4.9E-02	2.5E-06	6.9E-03	575
MEGA_SVO	IS_COV	4	167643601	169568082	1.2E-03	0.98	5.6E-05	2.5E-02	2.9E-07	3.1E-02	652
GIGA_CES	IS_COV	1	205459511	206705609	-4.5E-04	-0.95	7.3E-06	3.0E-02	3.7E-08	2.1E-02	180
SiGN_LAA	IS_COV	1	206706419	208158787	-2.4E-03	-0.74	3.5E-04	3.0E-02	1.4E-06	4.6E-02	401

chr: chromosome; start: start position of the genomic region from the input genome partition file; end: the end position of the genomic region from the input genome partition file; rho: the estimation of local genetic covariance; corr: the estimation of local genetic correlation; h2_1: the estimation of local heritability of the first trait; h2_2: the estimation of local heritability of the second trait; var: the variance of the estimation of local genetic covariance; p-value: the p-value of local genetic covariance; SNVs: number of single-nucleotide variants involved in the estimation of local genetic covariance in the genomic region.

Supplementary Table 4. SNPs with p-value <= 0.05 of the regions that are consistent in all analyses (p-value <= 0.05 and correlate in the same direction).

rsID	Chr	BP	A1	A2	Trait1	Trait2	p-value. Trait1	B.Trait1	SE.Trait1	p-value. Trait2	B.Trait2	SE.Trait2
**rs10033464**	4	111720761	T	G	GIGA_CES	IS-COV	1.4E-05	0.12	0.03	2.3E-02	0.04	0.02
**rs10033464**	4	111720761	T	G	MEGA_CES	IS-COV	1.5E-05	0.14	0.03	2.3E-02	0.04	0.02
**rs10033464**	4	111720761	T	G	SiGN_CES	IS-COV	9.8E-04	0.16	0.05	2.3E-02	0.04	0.02
**rs1006729**	2	129653290	A	G	GIGA_LAA	IS-COV	1.8E-03	-0.07	0.02	4.3E-02	0.02	0.01
**rs1006729**	2	129653290	A	G	MEGA_LAA	IS-COV	1.2E-03	-0.08	0.03	4.3E-02	0.02	0.01
**rs1006729**	2	129653290	G	A	SiGN_LAA	IS-COV	1.3E-03	0.11	0.04	4.3E-02	0.02	0.01
**rs10516563**	4	111677722	T	G	GIGA_CES	IS-COV	4.0E-43	-0.32	0.02	3.1E-02	-0.03	0.01
**rs10516563**	4	111677722	T	G	MEGA_CES	IS-COV	4.1E-36	-0.34	0.03	3.1E-02	-0.03	0.01
**rs10516563**	4	111677722	T	G	SiGN_CES	IS-COV	2.5E-16	-0.33	0.04	3.1E-02	-0.03	0.01
**rs1052536**	17	33331575	T	C	MEGA_LAA	IS-COV	3.9E-02	0.05	0.03	3.8E-02	0.02	0.01
**rs10853174**	17	33351917	T	C	MEGA_LAA	IS-COV	3.4E-02	0.05	0.03	1.7E-02	0.03	0.01
**rs10928853**	2	129708408	A	G	GIGA_LAA	IS-COV	2.3E-02	0.07	0.03	4.1E-02	0.03	0.01
**rs10928853**	2	129708408	G	A	SiGN_LAA	IS-COV	3.7E-02	-0.10	0.05	4.1E-02	0.03	0.01
**rs11930528**	4	111660194	T	G	GIGA_CES	IS-COV	7.1E-43	0.31	0.02	3.2E-02	-0.03	0.01
**rs11930528**	4	111660194	T	G	MEGA_CES	IS-COV	9.6E-37	0.34	0.03	3.2E-02	-0.03	0.01
**rs11930528**	4	111660194	G	T	SiGN_CES	IS-COV	1.3E-16	-0.33	0.04	3.2E-02	-0.03	0.01
**rs11931959**	4	111719685	A	G	GIGA_CES	IS-COV	1.4E-15	-0.14	0.02	1.9E-02	-0.03	0.01
**rs11931959**	4	111719685	A	G	MEGA_CES	IS-COV	1.2E-11	-0.14	0.02	1.9E-02	-0.03	0.01
**rs11931959**	4	111719685	A	G	SiGN_CES	IS-COV	7.7E-04	-0.11	0.03	1.9E-02	-0.03	0.01
**rs12451867**	17	32822800	T	C	GIGA_LAA	IS-COV	2.3E-02	0.07	0.03	2.3E-03	0.05	0.02
**rs12451867**	17	32822800	T	C	MEGA_LAA	IS-COV	4.9E-02	0.06	0.03	2.3E-03	0.05	0.02
**rs12503217**	4	111706161	T	C	GIGA_CES	IS-COV	2.1E-05	-0.12	0.03	4.4E-02	0.04	0.02
**rs12503217**	4	111706161	T	C	MEGA_CES	IS-COV	2.4E-05	-0.14	0.03	4.4E-02	0.04	0.02
**rs12503217**	4	111706161	T	C	SiGN_CES	IS-COV	1.6E-03	-0.15	0.05	4.4E-02	0.04	0.02
**rs12622701**	2	129663025	A	G	GIGA_LAA	IS-COV	3.7E-02	0.06	0.03	4.8E-02	0.03	0.01
**rs12622701**	2	129663025	G	A	SiGN_LAA	IS-COV	4.3E-02	-0.10	0.05	4.8E-02	0.03	0.01
**rs12644625**	4	111716513	T	C	GIGA_CES	IS-COV	1.7E-46	0.33	0.02	4.0E-02	-0.03	0.01
**rs12644625**	4	111716513	T	C	MEGA_CES	IS-COV	8.5E-39	0.35	0.03	4.0E-02	-0.03	0.01
**rs12644625**	4	111716513	C	T	SiGN_CES	IS-COV	1.9E-16	-0.33	0.04	4.0E-02	-0.03	0.01
**rs12646447**	4	111699326	T	C	GIGA_CES	IS-COV	6.1E-47	-0.33	0.02	3.9E-02	-0.03	0.01

**rs12646447**	4	111699326	T	C	MEGA_CES	IS-COV	1.0E-38	-0.35	0.03	3.9E-02	-0.03	0.01
**rs12646447**	4	111699326	T	C	SiGN_CES	IS-COV	9.6E-17	-0.33	0.04	3.9E-02	-0.03	0.01
**rs12941838**	17	32819326	A	G	GIGA_LAA	IS-COV	1.8E-02	0.07	0.03	3.6E-04	0.05	0.01
**rs12941838**	17	32819326	A	G	MEGA_LAA	IS-COV	2.8E-02	0.07	0.03	3.6E-04	0.05	0.01
**rs1446655**	2	129675712	T	C	GIGA_LAA	IS-COV	1.4E-02	-0.05	0.02	3.6E-02	0.02	0.01
**rs1446655**	2	129675712	T	C	MEGA_LAA	IS-COV	1.4E-03	-0.08	0.03	3.6E-02	0.02	0.01
**rs1446655**	2	129675712	C	T	SiGN_LAA	IS-COV	1.0E-03	0.12	0.04	3.6E-02	0.02	0.01
**rs16969891**	17	32796101	T	G	GIGA_LAA	IS-COV	5.2E-03	0.07	0.03	7.9E-03	0.04	0.01
**rs16969891**	17	32796101	T	G	MEGA_LAA	IS-COV	2.2E-03	0.09	0.03	7.9E-03	0.04	0.01
**rs16969896**	17	32798856	A	G	GIGA_LAA	IS-COV	5.3E-03	0.07	0.03	8.1E-03	0.04	0.01
**rs16969896**	17	32798856	A	G	MEGA_LAA	IS-COV	3.4E-03	0.08	0.03	8.1E-03	0.04	0.01
**rs16969940**	17	32821362	T	C	GIGA_LAA	IS-COV	3.0E-02	0.06	0.03	2.2E-03	0.05	0.02
**rs16969940**	17	32821362	T	C	MEGA_LAA	IS-COV	4.5E-02	0.06	0.03	2.2E-03	0.05	0.02
**rs17042088**	4	111654814	T	C	GIGA_CES	IS-COV	1.9E-42	0.31	0.02	4.2E-02	-0.03	0.01
**rs17042088**	4	111654814	T	C	MEGA_CES	IS-COV	5.2E-36	0.34	0.03	4.2E-02	-0.03	0.01
**rs17042088**	4	111654814	C	T	SiGN_CES	IS-COV	3.1E-16	-0.33	0.04	4.2E-02	-0.03	0.01
**rs17042121**	4	111677101	A	G	GIGA_CES	IS-COV	1.8E-44	-0.32	0.02	3.1E-02	-0.03	0.01
**rs17042121**	4	111677101	A	G	MEGA_CES	IS-COV	2.7E-37	-0.35	0.03	3.1E-02	-0.03	0.01
**rs17042121**	4	111677101	A	G	SiGN_CES	IS-COV	2.5E-16	-0.33	0.04	3.1E-02	-0.03	0.01
**rs17042144**	4	111689666	T	C	GIGA_CES	IS-COV	7.9E-45	-0.32	0.02	4.7E-02	-0.03	0.01
**rs17042144**	4	111689666	T	C	MEGA_CES	IS-COV	1.9E-37	-0.35	0.03	4.7E-02	-0.03	0.01
**rs17042144**	4	111689666	T	C	SiGN_CES	IS-COV	1.1E-16	-0.33	0.04	4.7E-02	-0.03	0.01
**rs17048931**	2	129740917	T	C	GIGA_LAA	IS-COV	4.4E-02	-0.06	0.03	3.9E-02	0.03	0.01
**rs17048931**	2	129740917	T	C	SiGN_LAA	IS-COV	4.5E-02	-0.09	0.05	3.9E-02	0.03	0.01
**rs2074518**	17	33324382	T	C	MEGA_LAA	IS-COV	3.6E-02	0.05	0.03	3.6E-02	0.02	0.01
**rs2074519**	17	33341834	T	C	GIGA_LAA	IS-COV	2.1E-02	-0.05	0.02	2.8E-02	-0.02	0.01
**rs2074519**	17	33341834	T	C	MEGA_LAA	IS-COV	2.2E-02	-0.06	0.03	2.8E-02	-0.02	0.01
**rs2100079**	2	129655682	A	G	GIGA_LAA	IS-COV	6.4E-04	-0.07	0.02	4.0E-02	0.02	0.01
**rs2100079**	2	129655682	A	G	MEGA_LAA	IS-COV	5.5E-04	-0.09	0.03	4.0E-02	0.02	0.01
**rs2100079**	2	129655682	A	G	SiGN_LAA	IS-COV	6.2E-04	-0.12	0.04	4.0E-02	0.02	0.01
**rs2220427**	4	111714889	T	C	GIGA_CES	IS-COV	6.2E-45	0.32	0.02	3.9E-02	-0.03	0.01
**rs2220427**	4	111714889	T	C	MEGA_CES	IS-COV	4.9E-37	0.34	0.03	3.9E-02	-0.03	0.01
**rs2220427**	4	111714889	C	T	SiGN_CES	IS-COV	2.4E-16	-0.33	0.04	3.9E-02	-0.03	0.01
**rs2220429**	4	111727485	A	C	GIGA_CES	IS-COV	4.2E-05	-0.11	0.03	2.3E-02	0.04	0.02
**rs2220429**	4	111727485	A	C	MEGA_CES	IS-COV	2.3E-05	-0.14	0.03	2.3E-02	0.04	0.02
**rs2220429**	4	111727485	C	A	SiGN_CES	IS-COV	8.3E-04	0.16	0.05	2.3E-02	0.04	0.02
**rs2246289**	2	129700617	T	C	GIGA_LAA	IS-COV	2.0E-03	0.07	0.02	2.3E-02	0.02	0.01
**rs2246289**	2	129700617	T	C	MEGA_LAA	IS-COV	7.0E-04	0.09	0.03	2.3E-02	0.02	0.01
**rs2246289**	2	129700617	T	C	SiGN_LAA	IS-COV	7.0E-04	0.12	0.04	2.3E-02	0.02	0.01
**rs2247701**	2	129663944	A	G	GIGA_LAA	IS-COV	2.0E-03	-0.07	0.02	4.4E-02	0.02	0.01
**rs2247701**	2	129663944	A	G	MEGA_LAA	IS-COV	1.7E-03	-0.08	0.03	4.4E-02	0.02	0.01
**rs2247701**	2	129663944	G	A	SiGN_LAA	IS-COV	9.9E-04	0.12	0.04	4.4E-02	0.02	0.01
**rs2247909**	2	129734206	A	C	GIGA_LAA	IS-COV	9.8E-04	0.07	0.02	2.3E-02	0.02	0.01
**rs2247909**	2	129734206	A	C	MEGA_LAA	IS-COV	4.3E-04	0.09	0.03	2.3E-02	0.02	0.01
**rs2247909**	2	129734206	A	C	SiGN_LAA	IS-COV	5.9E-04	0.12	0.04	2.3E-02	0.02	0.01
**rs2251354**	2	129728567	T	C	GIGA_LAA	IS-COV	4.9E-04	-0.08	0.02	3.6E-02	0.02	0.01
**rs2251354**	2	129728567	T	C	MEGA_LAA	IS-COV	2.2E-04	-0.09	0.03	3.6E-02	0.02	0.01
**rs2251354**	2	129728567	C	T	SiGN_LAA	IS-COV	4.3E-04	0.13	0.04	3.6E-02	0.02	0.01

**rs2254196**	2	129680903	A	C	GIGA_LAA	IS-COV	2.4E-03	-0.07	0.02	3.6E-02	0.02	0.01
**rs2254196**	2	129680903	A	C	MEGA_LAA	IS-COV	2.0E-03	-0.08	0.03	3.6E-02	0.02	0.01
**rs2254196**	2	129680903	C	A	SiGN_LAA	IS-COV	9.6E-04	0.12	0.04	3.6E-02	0.02	0.01
**rs2263458**	2	129691750	A	G	GIGA_LAA	IS-COV	8.4E-04	0.07	0.02	4.0E-02	0.02	0.01
**rs2263458**	2	129691750	A	G	MEGA_LAA	IS-COV	2.7E-04	0.09	0.03	4.0E-02	0.02	0.01
**rs2263458**	2	129691750	A	G	SiGN_LAA	IS-COV	6.9E-04	0.12	0.04	4.0E-02	0.02	0.01
**rs2264121**	2	129671244	A	G	GIGA_LAA	IS-COV	2.2E-03	0.07	0.02	3.6E-02	0.02	0.01
**rs2264121**	2	129671244	A	G	MEGA_LAA	IS-COV	1.9E-03	0.08	0.03	3.6E-02	0.02	0.01
**rs2264121**	2	129671244	A	G	SiGN_LAA	IS-COV	9.8E-04	0.12	0.04	3.6E-02	0.02	0.01
**rs2264824**	2	129688698	A	G	GIGA_LAA	IS-COV	8.9E-04	0.07	0.02	2.5E-02	0.02	0.01
**rs2264824**	2	129688698	A	G	MEGA_LAA	IS-COV	4.0E-04	0.09	0.03	2.5E-02	0.02	0.01
**rs2264824**	2	129688698	A	G	SiGN_LAA	IS-COV	9.1E-04	0.12	0.04	2.5E-02	0.02	0.01
**rs2406642**	2	129792155	T	C	SiGN_LAA	IS-COV	2.9E-02	-0.08	0.04	1.5E-02	-0.03	0.01
**rs2465821**	2	129717684	A	G	GIGA_LAA	IS-COV	7.2E-04	-0.07	0.02	2.4E-02	0.02	0.01
**rs2465821**	2	129717684	A	G	MEGA_LAA	IS-COV	3.9E-04	-0.09	0.03	2.4E-02	0.02	0.01
**rs2465821**	2	129717684	G	A	SiGN_LAA	IS-COV	6.0E-04	0.12	0.04	2.4E-02	0.02	0.01
**rs3135967**	17	33313729	A	G	MEGA_LAA	IS-COV	2.7E-02	-0.05	0.02	1.7E-02	0.02	0.01
**rs4032974**	4	111732536	T	C	GIGA_CES	IS-COV	2.3E-05	-0.11	0.03	2.3E-02	0.04	0.02
**rs4032974**	4	111732536	T	C	MEGA_CES	IS-COV	2.0E-05	-0.14	0.03	2.3E-02	0.04	0.02
**rs4032974**	4	111732536	C	T	SiGN_CES	IS-COV	1.2E-03	0.16	0.05	2.3E-02	0.04	0.02
**rs4124159**	4	111730243	A	G	GIGA_CES	IS-COV	7.3E-06	0.12	0.03	2.3E-02	0.04	0.02
**rs4124159**	4	111730243	A	G	MEGA_CES	IS-COV	8.8E-06	0.14	0.03	2.3E-02	0.04	0.02
**rs4124159**	4	111730243	A	G	SiGN_CES	IS-COV	1.2E-03	0.16	0.05	2.3E-02	0.04	0.02
**rs4331451**	2	129696104	A	G	GIGA_LAA	IS-COV	4.1E-02	0.06	0.03	4.1E-02	0.03	0.01
**rs4331451**	2	129696104	G	A	SiGN_LAA	IS-COV	3.9E-02	-0.10	0.05	4.1E-02	0.03	0.01
**rs4605724**	4	111685081	A	C	GIGA_CES	IS-COV	1.5E-43	0.32	0.02	4.7E-02	-0.03	0.01
**rs4605724**	4	111685081	A	C	MEGA_CES	IS-COV	1.9E-36	0.34	0.03	4.7E-02	-0.03	0.01
**rs4605724**	4	111685081	C	A	SiGN_CES	IS-COV	1.4E-16	-0.33	0.04	4.7E-02	-0.03	0.01
**rs4626276**	4	111649989	A	C	GIGA_CES	IS-COV	4.9E-42	-0.31	0.02	4.2E-02	-0.03	0.01
**rs4626276**	4	111649989	A	C	MEGA_CES	IS-COV	2.8E-36	-0.34	0.03	4.2E-02	-0.03	0.01
**rs4626276**	4	111649989	A	C	SiGN_CES	IS-COV	3.4E-16	-0.33	0.04	4.2E-02	-0.03	0.01
**rs4662630**	2	129773352	C	T	SiGN_LAA	IS-COV	2.7E-02	-0.08	0.04	1.3E-03	-0.04	0.01
**rs7583520**	2	129795079	C	T	SiGN_LAA	IS-COV	2.9E-02	-0.08	0.04	1.2E-02	-0.03	0.01
**rs797989**	17	33414758	A	C	MEGA_LAA	IS-COV	3.8E-02	0.05	0.03	1.0E-02	0.03	0.01

Chr: chromosome; BP: base pair position; A1: effect allele; A2: alternative allele; p-value.Trait.: the p-value of the single-nucleotide variants in the GWAS; B.Trait: the effect calculated with the effect allele in the GWAS; SE.Trait: standard error.

References

1. Chung, J.W.; Park, S.H.; Kim, N.; Kim, W.J.; Park, J.H.; Ko, Y.; Yang, M.H.; Jang, M.S.; Han, M.K.; Jung, C.; et al. Trial of ORG 10172 in Acute Stroke Treatment (TOAST) Classification and Vascular Territory of Ischemic Stroke Lesions Diagnosed by Diffusion-Weighted Imaging. *J. Am. Heart Assoc.* **2014**, *3*, e001119. https://doi.org/10.1161/JAHA.114.001119.
2. Mishra, A.; Malik, R.; Hachiya, T.; Jürgenson, T.; Namba, S.; Posner, D.C.; Kamanu, F.K.; Koido, M.; le Grand, Q.; Shi, M.; et al. Stroke Genetics Informs Drug Discovery and Risk Prediction across Ancestries. *Nature* **2022**, *611*, 115–123. https://doi.org/10.1038/s41586-022-05165-3.
3. Malik, R.; Chauhan, G.; Traylor, M.; Sargurupremraj, M.; Okada, Y.; Mishra, A.; Rutten-Jacobs, L.; Giese, A.K.; Van Der Laan, S.W.; Gretarsdottir, S.; et al. Multiancestry Genome-Wide Association Study of 520,000 Subjects Identifies 32 Loci Associated with Stroke and Stroke Subtypes. *Nat. Genet.* **2018**, *50*, 524–537. https://doi.org/10.1038/s41588-018-0058-3.

4. A First Update on Mapping the Human Genetic Architecture of COVID-19. *Nature* **2022**, *608*, E1–E10. https://doi.org/10.1038/S41586-022-04826-7.
5. Budde, M.; Anderson-Schmidt, H.; Gade, K.; Reich-Erkelenz, D.; Adorjan, K.; Kalman, J.L.; Senner, F.; Papiol, S.; Andlauer, T.F.M.; Comes, A.L.; et al. A Longitudinal Approach to Biological Psychiatric Research: The PsyCourse Study. *Am. J. Med. Genet. B. Neuropsychiatr. Genet.* **2019**, *180*, 89–102. https://doi.org/10.1002/AJMG.B.32639.
6. Bycroft, C.; Freeman, C.; Petkova, D.; Band, G.; Elliott, L.T.; Sharp, K.; Motyer, A.; Vukcevic, D.; Delaneau, O.; O'Connell, J.; et al. The UK Biobank Resource with Deep Phenotyping and Genomic Data. *Nature* **2018**, *562*, 203–209. https://doi.org/10.1038/S41586-018-0579-Z.
7. Domingues-Montanari, S.; Fernández-Cadenas, I.; del Río-Espinola, A.; Mendioroz, M.; Fernandez-Morales, J.; Corbeto, N.; Delgado, P.; Ribó, M.; Rubiera, M.; Obach, V.; et al. KCNK17 Genetic Variants in Ischemic Stroke. *Atherosclerosis* **2010**, *208*, 203–209. https://doi.org/10.1016/J.ATHEROSCLEROSIS.2009.07.023.
8. Fernández-Cadenas, I.; Mendióroz, M.; Giralt, D.; Nafria, C.; Garcia, E.; Carrera, C.; Gallego-Fabrega, C.; Domingues-Montanari, S.; Delgado, P.; Ribó, M.; et al. GRECOS Project (Genotyping Recurrence Risk of Stroke): The Use of Genetics to Predict the Vascular Recurrence After Stroke. *Stroke* **2017**, *48*, 1147–1153. https://doi.org/10.1161/STROKEAHA.116.014322.
9. Riba, I.; Jarca, C.I.; Mundet, X.; Tovar, J.L.; Orfila, F.; Nafría, C.; Raga, A.; Girona, A.; Fernández-Lara, P.; Castañé, X.; et al. Cognitive Assessment Protocol Design in the ISSYS (Investigating Silent Strokes in HYpertensives: A Magnetic Resonance Imaging Study). *J. Neurol. Sci.* **2012**, *322*, 79–81. https://doi.org/10.1016/J.JNS.2012.06.015.
